# Classification and Quantification of Unproductive Splicing Events

**DOI:** 10.32607/actanaturae.27572

**Published:** 2025

**Authors:** L. G. Zavileyskiy, E. A. Chernyavskaya, M. A. Vlasenok, D. D. Pervouchine

**Affiliations:** Center for Molecular and Cellular Biology, Skolkovo Institute of Science and Technology, Moscow, 121205 Russia

**Keywords:** unproductive splicing, nonsense mediated decay, NMD, splicing, regulation

## Abstract

In eukaryotic cells, the nonsense-mediated decay (NMD) pathway degrades mRNAs with premature stop codons. The coupling between NMD and alternative splicing (AS) generates NMD-sensitive transcripts (NMD targets, NMDTs) that play an important role in the gene expression regulation via the unproductive splicing mechanism. Understanding this mechanism requires proper identification of NMDT-generating AS events. Here, we developed NMDj, a tool for the identification, classification and quantification of NMDTgenerating AS events which does not rely on the best matching transcript partner principle employed by the existing methods. Instead, NMDj uses a set of characteristic introns that discriminate NMDTs from all protein-coding transcripts. The benchmark on simulated RNA-Seq data demonstrated that NMDj allows to quantify NMDT-generating AS events with better precision compared to other existing methods. NMDj represents a generic method suitable for the accurate classification of arbitrarily complex AS events that generate NMDTs. The NMDj pipeline is available through the repository https://github.com/zavilev/NMDj/.

## INTRODUCTION

Eukaryotic cells express a large number of transcripts from each gene through alternative splicing (AS). By rough estimates, human protein-coding genes produce as many as ~150,000 expressed transcripts, an average of 7.4 isoforms per gene [1]. However, only half of these transcripts encode full-length proteins, while the remaining part may contain premature termination codons (PTC) [1, 2]. In eukaryotes, such transcripts are selectively eliminated by the pathway called the nonsense-mediated decay (NMD) [3].

In recent studies, it has been proposed that NMD not only prevents the translation of truncated proteins resulting from nonsense mutations and splicing errors, but is also involved in a wide range of biological processes, including gene expression regulation [4]. Most RNA-binding proteins (RBPs) control their own expression levels through a negative feedback loop in which the gene product binds to its cognate mRNA and induces AS that generates a PTC [5, 6]. It has been suggested that much of the impact of AS on the eukaryotic transcriptional landscape is mediated by the generation of NMD isoforms to limit gene expression, rather than the expansion of proteome diversity [2].

Local splicing changes, that is, the ones confined to a local region in the pre-mRNA, are one of the main sources of transcripts that are NMD targets (NMDT). Among the main types of local AS events, one can distinguish the so-called poison and essential exons which lead to the generation of NMDT upon exon inclusion and skipping, respectively, as well as the use of alternative 5’- and 3’-splice sites and intron retention [7]. Some of them (for instance, intron retention) may be involved in a particular biological process or may be preferentially regulated by the same splicing factor [8, 9]. However, the diversity of AS events is not limited to the main types listed above [6]. The task of characterizing complex AS events leading to the emergence of NMDT appears in many studies related to gene expression regulation [10, 11, 12].

To date, the only solution to this problem has been provided by the NMD Classifier [13]. Its approach is based on the assumption of minimal evolution/regulation, according to which NMDTs are the result of evolutionary or regulatory events that alter minimally the reading frame of a protein-coding transcript. That is, NMD Classifier finds the most similar coding transcript (in terms of shared nucleotide sequence) for each NMDT and considers the differences between the best partner transcript and NMDT which cause a frameshift to be the generating AS event. However, the probability of NMDT being derived from a protein- coding transcript via AS depends not only on the similarity in their exon-intron architectures but also on their expression levels. The coding transcript with the highest expression level is more likely to be the source of NMDT [14]. Furthermore, NMDT may be derived from different transcripts with comparable expression levels, which calls into question the validity of the approach based on the selection of only one matching transcript partner.

In revisiting this problem, we developed NMDj, a tool for systematic search, classification and quantification of NMDT-generating AS events which takes into account all annotated transcripts and reports all introns that distinguish NMDTs from protein-coding transcripts. NMDj provides a more detailed classification of NMDT-generating AS events than the NMD Classifier. The coupling between NMD and AS is a crucial post-transcriptional mechanism of gene expression regulation [15]. Therefore, developing a method for searching, classifying, and quantifying AS events leading to NMDT which would take into account all the diversity of transcript isoforms is challenging. The NMDj method is aimed at tackling exactly this problem. It receives a set of transcripts in the form of an annotation database or transcript models constructed from RNA sequencing data as input, and provides the characterization of NMDTgenerating AS events and their quantification as output.

## EXPERIMENTAL

**Genome annotation**

The annotations of the human (GRCh38, version 108), mouse (mm10, version 113), zebrafish (danRer11, version 113), and Drosophila (dm6, version 113) genomes were downloaded from Ensembl in GTF format [16]. Only the transcripts of protein-coding genes with at least one annotated NMDT were considered. Transcripts without an annotated start or stop codon were filtered out. Genes without either NMDT or protein- coding transcripts were not considered.

**NMD Classifier**

The NMD Classifier source code was downloaded from [13]. To quantify local splicing alterations, the NMD Classifier output was converted to a list of alternative splice junctions corresponding to the four main types of AS events: alternative exons, alternative 5’- and 3’-splice sites, and intron retention (NMD_in, NMD_ex, A5SS, A3SS, NMD_IR, nNMD_IR).

**The NMDj pipeline**

The pipeline departs from a transcript annotation file in GFF/GTF format [17]. The following four features (”transcript”, ”exon”, ”start_codon” and ”stop_ codon”) and three attributes (”gene_id”, ”transcript_ id”, ”transcript_type”) are considered. In addition to the main GFF/GTF file, NMDj can also accept a secondary input containing “transcript” and “exon” features, along with the “transcript_id” attribute. In this case, each transcript from the additional file is assigned to a gene from the main file based on the maximum number of common introns and a sequence overlap of at least 50%. For transcripts that were assigned to genes, the longest open reading frame is selected from those containing the annotated start codons and the corresponding start and stop codon positions are added to the annotation. As in Ensembl [18], a transcript is annotated as NMDT if there is an intron at least 50 nt downstream of the stop codon position.

Next, for each NMDT, NMDj considers the genomic interval spanning from the last splice site shared by NMDT and any protein-coding transcript with the same phase, or start codon in the absence of such, to the 3’end of the exon with PTC, or the closest downstream transcript end, if NMDT shares its stop codon with a protein-coding transcript. The characteristic introns are defined as all introns overlapping the genomic interval of interest except those shared by the NMDT and any coding transcript. The NMDTgenerating AS event is defined as the set of characteristic introns described above. AS events from a pair of NMDTs are merged into a cluster if the NMDTs share at least one characteristic intron.

To classify NMD-generating AS events, NMDj by default uses MANE-Select transcripts as a reference, since they tend to be the most expressed ones [19]. However, a user-defined input can also be provided. NMDj builds a directed acyclic splicing graph using the splice sites of NMDT and splice sites of the reference transcript as nodes and introns and exons as edges, and it searches for “bubbles” defined by vertex-independent paths that contain characteristic introns [20, 21]. NMDj reports all found pairs of vertex-independent paths in the following form: X_1…_X_n_ : Y1_…_Y_m_, where X_i_ and Y_j_ are “D” (donor) and “A” (acceptor) symbols, and X_i_≠X_j_ and Y_i_≠Y_j_ when j = i ± 1. If the reference transcript set has not been specified, then NMDj iteratively compares the NMDT with each protein-coding transcript.

The last, optional step is the quantification of AS events using RNA-seq split read counts (the input table must be provided). NMDj computes the Ψ (percent- spliced-in) values, which estimate the expression level of the NMDT relative to all the transcripts of the gene. It is calculated using the formula

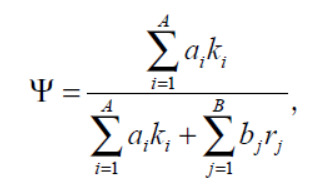



where *A *and *B *are the number of characteristic introns supporting NMDT and protein-coding transcripts, respectively; *a_i_*and *b_j_*are the number of RNAseq split-reads aligned to the respective introns, and* k_i_*and *r_j_*are the weights that account for the number of times the characteristic introns occur in NMDT and coding transcripts, respectively. The weights *ki *and *rj* are computed independently for NMDT and coding transcripts. The natural requirement that the sum of the weights of the characteristic introns of each transcript be equal to 1 leads us to a system of *n *linear equations with *m *unknowns, where *n *is the number of transcripts and *m *is the number of characteristic introns. By the construction of characteristic introns, such a system is always consistent, but it can also have an infinite number of solutions. In general, one could make an unambiguous choice of *ki *and *rj* by imposing regularization constraints on this system. However, in NMDj we use the following heuristic algorithm, which allows us to define the value of Ψ in accordance with the existing definitions for the main types of AS events [6, 11].

Transcripts annotated in the interval are represented as a graph with the vertices being characteristic introns, and the edges being the exons (or their groups) that connect them. This graph is searched for pairs of vertices connected by only vertex-independent paths. For each such path, the weights of characteristic introns are assumed to be equal to each other. For a poison exon, for instance, there will be two such paths: one corresponding to exon inclusion (with two characteristic introns, each with a weight of 0.5), and another corresponding to exon skipping (with one characteristic intron, the weight of which is equal to 1). After the coefficients of the nodes between the identified pair are assigned values, these nodes are merged into one and the search in the new graph continues. At each step, the coefficients of the characteristic introns combined into a node are multiplied by the value assigned to that node and the procedure continues until all nodes are merged into one. This algorithm works for all simple types of AS events, and for complex AS events it works only under the assumption that all vertex-independent paths are nested.

**The real and simulated RNA-seq data**

To realistically model RNA sequencing data using known transcript expression levels, and hence relative NMDT expression levels, we selected three random samples in each of the three tissues (Muscle, Liver, and Cerebellum) using the panel of transcriptomic data from the Genotype-Tissue Expression project (GTEx [22]. The choice of the tissues was motivated by the fact that they differ most drastically in terms of AS [23, 24]. Transcript expression levels in the selected samples were obtained by rsem-calculate- expression with the --estimate-rspd option [25]. The expression levels of NMDTs, best partner transcripts, and MANE-Select transcripts as a fraction of the total gene expression were calculated for each gene. Sampling was repeated five times, and the results were averaged.

RNA-seq data simulation was performed by rsemsimulate- reads based on the transcript expression levels described above. For each sample, 50 mln pairedend reads were simulated. The simulated reads were aligned to the GRCh38 human genome using STAR aligner 2.7.3a [26]. Counts of split-reads were obtained using the IPSA package with default settings [27]. Transcript expression levels in the simulated samples were quantified by RSEM (as above) [25]; Salmon 1.10.3, with the options --seqBias --gcBias --posBias [28]; and StringTie 2.2.3, with the option -e [29]. To convert transcript-level quantification results to Ψ values of the AS events, the NMDT expression levels (in TPM, transcripts per million) were divided by the sum of expression levels of transcripts spanning the genomic regions found by NMDj.

**RNA-seq data on NMD inactivation**

The results of the experiments on the inactivation of NMD components (double knockdown of SMG6 and SMG7) followed by RNA-seq were obtained from Gene Expression Omnibus under the accession number GSE86148 in the FASTQ format and aligned to the human genome assembly GRCh38 (hg38) using the STAR aligner v2.7.8a in the paired-end mode. Counts of split-reads were obtained using the IPSA package in the default settings [27].

## RESULTS

**The NMDj pipeline**

**Fig. 1:**
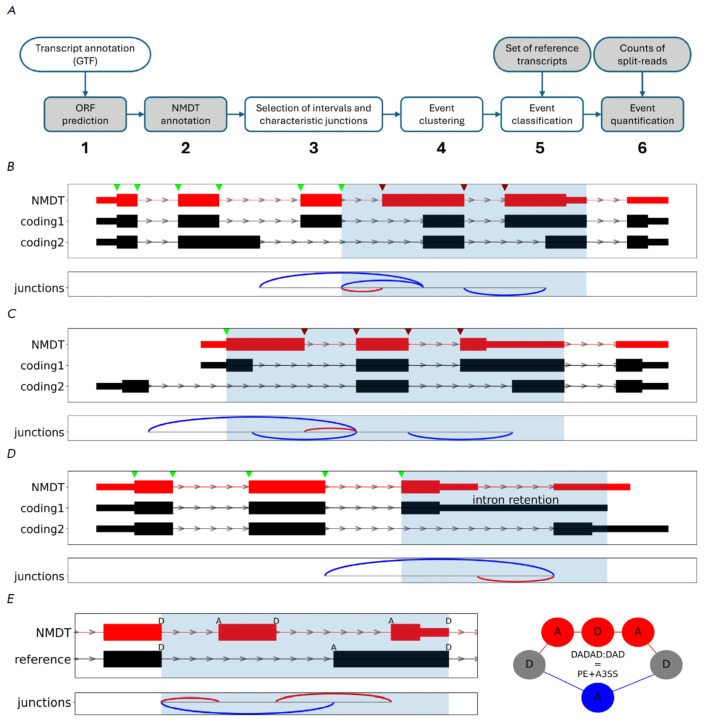
The NMDj pipeline. (A) The pipeline flowchart. (B–D) The choice of interval boundaries (light blue shading). The 5’-boundary is either the last splice site common to NMDT and any coding transcript with the same phase (B), or the start codon if there is no such splice site (C). The 3’-boundary is either the donor splice site of the intron following the PTC-containing exon (B, C), or the end of the shortest 3’-UTR downstream of the NMDT stop codon (D). (E) An example of classification based on vertex-independent paths. NMDT and its reference coding transcript (left) correspond to a pair of vertex-independent paths consisting of donor and acceptor splice sites (right). NMDT and protein-coding transcripts, as well as their corresponding characteristic introns (arcs), are shown in red and blue, respectively. Splice sites of NMDT are indicated by green arrows if the NMDT frame matches the protein-coding frame, or red otherwise

The NMDj pipeline consists of three main and three auxiliary steps (*Fig. 1A*). Starting from the transcript annotation database, it performs the reading frame search and predicts NMDT, if they are not annotated. NMDT are annotated based on the so-called 50-nt rule, which postulates that a transcript is recognized as an NMD target if it contains an intron at least 50–55 nt downstream of the stop codon [30]. This rule departs from the assumption that exon junction complexes that are deposited on pre-mRNA during splicing are displaced during the pioneer round of translation, and ones that remain bound outside of the reading frame serve as a PTC signal [30]. In NMDj, we used the threshold of 50 nucleotides because this is the accepted value for automatic NMDT annotation in Ensembl [16]. However, the number of predicted NMDTs changes insignificantly when the threshold is increased to 55 nt (*Fig. S1*).

Once open reading frames are detected and NMDTs are annotated for each gene, NMDj begins searching for NMD-generating AS events. There exist multiple formalisms for describing AS events including binary classes (such as poison exons [31]), classification of connected components in a splice graph [32], and local splicing variations [33]. In this work, we define an AS event as a set of characteristic introns spanning the following genomic interval. For each NMDT, the 5’-boundary of the interval is defined to be the 3’-most splice site, which it shares with any protein-coding transcript with the same phase (*Fig. 1B*). If no such splice site exists, the 5’ boundary is placed at the start codon of NMDT, if it is shared with at least one protein-coding transcript (*Fig. 1C*). The 3’-boundary of the interval is defined to be the 3’-end of the PTC-containing exon or, if NMDT shares its stop-codon with a protein-coding transcript, and in case it is not a true PTC, it is placed at the nearest transcript end (*Fig. 1D*).

Next, NMDj selects the characteristic introns that distinguish NMDT from protein-coding transcripts. All the introns that are adjacent to the interval or overlap with it, except the ones that are shared by the NMDT and at least one protein-coding transcript, are considered to be characteristic introns. As a result, each NMDT is characterized by a set of characteristic introns that originate either from it or from proteincoding transcripts (*Fig. 1B,D*, red and blue arcs). The characteristic introns are merged into clusters to reduce redundancy, as several NMDTs would often possess the same or very similar sets of characteristic introns.

NMDj classifies splicing events into major types such as poison (PE) and essential (EE) exons, alternative splice sites (A5SS, A3SS), and others (*Table 1*). The classification of the AS events is based on the concept of vertex-independent paths applied to splicing graphs [20, 34]. In a directed acyclic graph, whose nodes are donor (D) and acceptor (A) splice sites, and edges are exons and introns, one can define a vertexindependent path as a pair of paths that do not share any nodes except the first and last node (*Fig. 1E*). Each such pair is reported in a symbolic form representing the sequence of nodes; i.e., a poison exon (PE) corresponds to DADA:DA; an alternative 5’- splicesite (A5SS), to ADA:ADA; and multiple poison exons (PEn), to D(AD)nA:DA, where *n *is the number of exons. In the final step, NMDj quantifies each group of NMDTs by Ψ values based on split read counts from RNA-Seq experiments (see EXPERIMENTAL).

**Table 1:** A list of NMDj event types and their synonyms in a classification provided by NMD Classifier

Type	NMDj	Description	Synonym
DADA:DA	PE	Poison cassette exon which triggers NMD upon inclusion	NMD_in
D(AD)nA:DA	PEn	n consecutive cassette exons which trigger NMD upon simultaneous inclusion	multi_NMD_in
DA:DADA	EE	Essential cassette exon which triggers NMD upon skipping	NMD_ex
DA:D(AD)nA	EEn	n consecutive cassette exons which trigger NMD upon simultaneous skipping	multi_NMD_ex
ADA:ADA	A5SS	Alternative 5’-splice sites	A5SS
DAD:DAD	A3SS	Alternative 3’-splice sites	A3SS
ADAD:ADAD	A5SS+A3SS	Both 5’- and 3’-splice sites of the same intron are alternative	A5SS,A3SS
AD:ADAD	IR	Intron retention which triggers NMD	nNMD_IR
ADAD:AD	ID	Intron excision which triggers NMD	NMD_IR
DADA:DADA	MXE	A pair of mutually exclusive adjacent exons	-
AD(AD)nA:ADA	A5SS+PEn	Alternative 5’-splice site and n consecutive poison exons	-
ADA:AD(AD)nA	A5SS+EEn	Alternative 5’-splice site and n consecutive essential exons	-
D(AD)nAD:DAD	PEn+A3SS	n consecutive poison exons and alternative 3’-splice site	-
DAD:D(AD)nAD	EEn+A3SS	n consecutive essential exons and alternative 3’-splice site	-
ADAD:AD(AD)nAD	A5SS+EE+A3SS	Alternative 5’-splice site, n consecutive essential exons and alternative 3’-splice site	-

**NMDj in application to human and model organism transcripts**

The application of NMDj to annotated transcripts from human, mouse, zebrafish, and Drosophila showed that the proportion of NMDTs obeying the 50-nucleotide rule is significantly higher in humans and mice than it is in zebrafish and Drosophila, which is undoubtedly a result of differences in the quality and completeness of transcriptome annotations (*Table 2*). However, the frequencies of NMDT-generating AS events vary significantly between organisms. While in humans and mice NMDTs are generated more frequently through the use of poison and essential exons than they are through intron retention, in Drosophila and zebrafish the pattern is opposite. According to existing estimates, the proportion of intron retention among the major AS types is equally low in mammals as it is in other vertebrates and invertebrates [35]. Thus, the observed difference between NMDTgenerating AS event frequencies can be explained neither by the different levels of abundance of their types nor by the different levels of completeness of the transcriptome annotation. Rather, the difference indicates the peculiarities of the NMD system’s functioning in different taxonomic groups.

**Table 2:** NMD-generating AS events in the human and model organism’s transcriptomes

	#Tr	#NMDT	NMDT, %	Fraction of AS events, %					
				PE	EE	A5SS	A3SS	IR	Other
Human	79940	16741	21	18	11	6	8	2	55
Mouse	49951	5339	11	18	18	11	14	4	36
Zebrafish	35040	854	2	11	10	11	12	23	32
Drosophila	30688	1325	4	18	4	12	9	16	41

Note: #Tr – total number of transcripts;

#NMDT – number of NMDT;

NMDT – fractions of NMTD (in %). Fractions

(in %) of toxic (PE) and necessary (EE) exons, fractions of alternative 5’-(A5SS) and 3’-splicing sites (A3SS), fractions of retained introns (IR) and other events (Other).

**The advantages of NMDj in finding NMD-generating AS events**

The existing approach to the analysis of NMDgenerating AS events, which is implemented in the NMD Classifier, is based on choosing the best partner transcript. The main problem in this approach is that other transcripts and their expression levels are not taken into account when selecting the best partner transcript. A protein-coding transcript is unlikely to be the main source of NMDT if its expression level is low. To illustrate the importance of this issue, we applied the NMD Classifier to the Ensembl transcriptome annotation [16] and compared the identified set of best partner transcripts with those from the MANE-Select annotation considered as the set of the most expressed transcripts in each human gene [19].

**Fig. 2:**
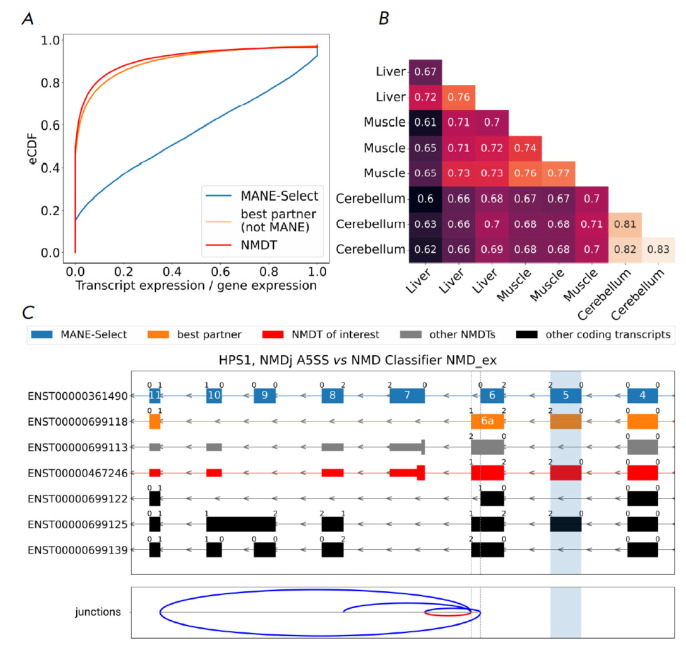
NMDj and NMD Classifier best partner transcripts. (A) Transcript relative abundance (eCDF is the cumulative distribution function) estimated from a random sample of RNA-seq experiments from GTEx. (B) The proportion of genes whose most expressed transcripts match between pairs of GTEx tissue samples. (C) An example of a local NMD-generating event in the HPS1 gene. The characteristic introns originating from NMDTs and protein-coding transcripts are shown by red and blue arcs, respectively. The phase of the reading frame is indicated above the exon boundaries. Transcript colors: MANE-Select (blue), NMDT (red), best partner transcript from NMD Classifier (orange), other transcripts (gray – NMDT, black — protein-coding). The essential exon predicted by the NMD classifier is highlighted in light-blue; however, NMDT is actually generated by a splice site shift in the MANE-Select isoform

MANE-Select transcripts were identified as best partners only for 25% of NMDTs, while they had a significantly higher expression level, as confirmed by a random sample of RNA-seq experiments from GTEx (*Fig. 2A*). Furthermore, when the best partner transcript was not MANE-Select, its contribution to the total gene expression level was comparable to that of NMDT. This suggests that the transcript that is most similar to NMDT in terms of the shared sequence can be at the same time a poor candidate for generating NMDT. Moreover, the MANE-Select transcripts are not always the most expressed ones. Tissues may differ in their most expressed transcripts (*Fig. 2B*) or express several transcripts at comparable levels. To address this, NMDj considers all annotated transcripts in order to avoid the problem of choosing one best transcript partner and clusters NMDTs with similar characteristic introns to obtain a concise and non-redundant set of AS events (*Fig. 2D*).

NMDj is particularly useful in genes with a complex splicing architecture. A notable example is *HPS1*, which contains a group of exons with lengths that are not multiples of three (*Fig. 2C*). Skipping of each single exon generates a NMDT, unless it is compensated by a downstream AS event that restores the coding frame. Simultaneous inclusion of exons 6a and 7 generates a NMDT. NMD Classifier selects the transcript with exon 5 as the best partner. This exon is skipped in the NMDT, which indeed disrupts the coding frame. However, it is also skipped in a proteincoding transcript, in which its frameshift is compensated by using exon 6 instead of exon 6a and skipping exons 7–10. NMDj correctly identifies the last splice site, in which the reading frame of NMDT matches that of a coding transcript, to be the 3’-boundary of exon 6a, which enables the detection of the only true NMD-generating AS event; namely, the splice junction between exons 6a and 7. It also identifies all alternative introns whose excision helps to bypass frame shifts. Interestingly, another NMDT with exon 5 included shares a characteristic intron with the previous one and is therefore clustered with it by NMDj.

**NMDj provides a more detailed AS event classification**

**Fig. 3:**
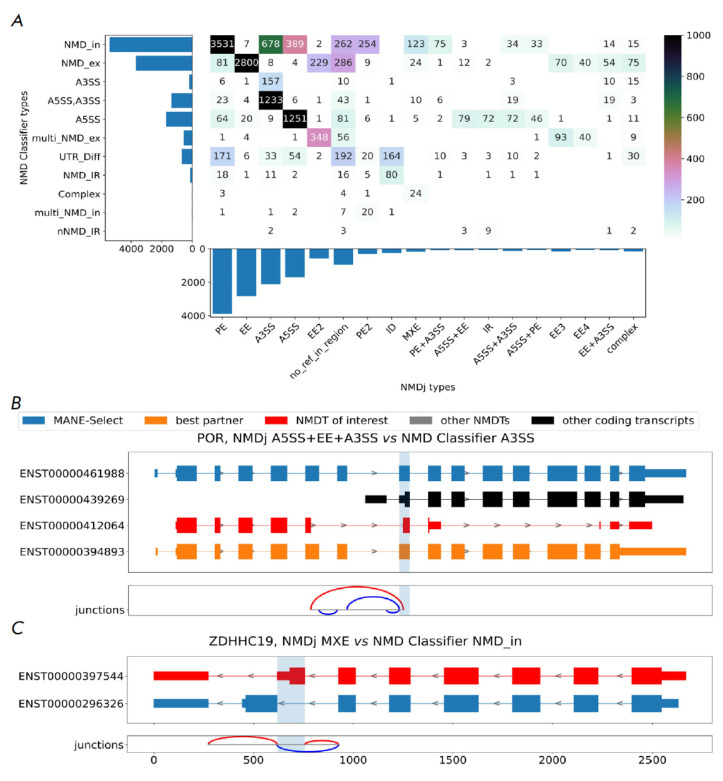
AS event categorization. (A) A comparison of classifications by NMDj and NMD Classifier. Each cell represents the number of NMDTs classified into corresponding types by NMDj (rows) and NMD Classifier (columns). (B, C) Examples of rare NMD-generating AS events. The rest of the legend is as in Fig. 2

We compared the classification of AS events produced by NMDj and NMD Classifier in application to the same human transcriptome annotation (*Fig. 3A*). NMDj was configured to use MANE-Select transcripts as a reference. Out of 15,914 NMDTs, NMD Classifier and NMDj were able to classify AS events for 15,446 and 15,265 NMDTs, respectively. However, AS events were classified into the same type (*Table 1*) for only 60% of NMDTs.

While NMD Classifier subdivides AS events into a fixed number of most common types, NMDj is able to describe more complex splicing patterns using vertex- independent paths. In the *POR *gene, for example, NMDT differs from protein-coding isoforms by alternative 5’- and 3’-splice sites and a cassette exon (*Fig. 3B*). Such events tend to evade many standard tools for splicing analysis [31, 32]. The presence of AS types, which NMD Classifier is unable to properly detect, accounts for a large portion of inconsistencies between the two classifications. For example, a number of events classified by NMD Classifier as poison exons (NMD_in) are classified as PE+A3SS and MXE by NMDj (*Fig. 3 A,C*). Another advantage of NMDj is the ability to classify AS events in 3’-untranslated regions (3’-UTRs). Among the events that induce NMD in the 3’-UTRs, the majority are expectedly represented by intron retention. Moreover, many 3’-UTR events do not intersect with the MANE-Select isoform (*Fig. 3A, S2*).

A relatively small number of other inconsistencies may be explained by the fact that NMDj and NMD Classifier use different reference transcripts to classify AS events. Only 61% of AS were classified in the same type when NMDj was configured to use best partner transcripts as a reference. A substantial portion of the differences seems to be the result of misclassification by NMD Classifier. For example, most events attributed to the “A3SS, A5SS” type by NMD classifier are classified as A3SS by NMDj (*Fig. 3A*). Meanwhile, the size of the NMD classifier’s “A5SS,A3SS” class is far larger than the size of the “A3SS” class. This is counterintuitive, since the choice between a pair of alternative 5’-splice sites seems to be independent from the choice between a downstream pair of 3’-splice sites separated by a long intron [36]. Visual inspection of randomly selected individual cases of classification discrepancy confirmed the correctness of the classification provided by NMDj (*Fig. S3*).

**NMDj benchmark on simulated and real data**

**Fig. 4:**
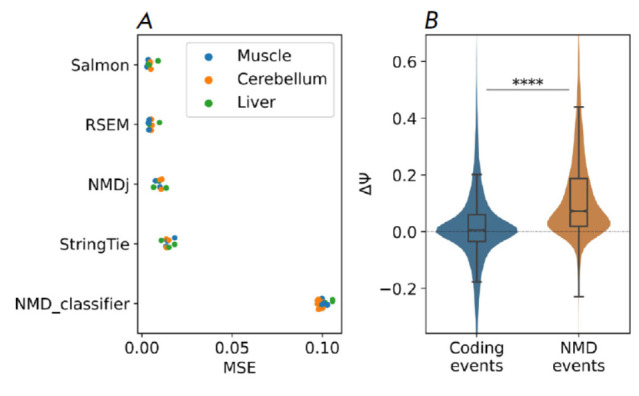
Comparison of NMDj and NMD Classifier predictions. (A) Mean squared error (MSE) of Ψ values estimated by different methods from simulated RNA-Seq data relative to the ground truth values. (B) Statistically significant splicing changes of NMDT-generating and non-NMDT protein-coding AS events (cassette exons, alternative splice sites and intron retention) upon NMD inactivation by cycloheximide, quantified by NMDj. **** denotes statistically significant differences at the 0.1% significance level (Mann–Whitney test)

NMD-generating AS events can be used to assess relative NMDT expression levels quantitatively using RNA-seq data. To evaluate the accuracy of NMDj in quantifying AS, we simulated RNA-seq reads based on the average transcript expression levels in human tissues. The estimated Ψ values computed from split reads aligned to characteristic introns were compared to the ground truth Ψ values, defined as the NMDT isoform abundance as a fraction of the total abundance of all transcripts of the given gene. As a measure of distance, we used the mean squared error (MSE) over all Ψ values across all the NMDT isoforms tested. It turned out that NMDj performed comparably to existing state-of-the-art methods for transcript-level quantification, while the MSE values for NMD classifier were substantially larger (*Fig. 4A*). Since the methods used to calculate the Ψ metric in NMDj and NMD Classifier were identical, this again suggests that not only the best partner transcript but also other transcripts contribute significantly to the Ψ value.

To confirm that AS events predicted by NMDj actually generate NMDT, we compared the changes in the Ψ values of AS events generating and not generating NMDTs in NMD inactivation experiments implementing knockdown of its two key factors: SMG6 and SMG7 [14]. AS events that did not generate NMDT included cassette exons, alternative splice sites, and retained introns that had been found in non-NMDT protein-coding transcripts. As expected, upon inactivation of NMD, the Ψ values of NMD-generating AS events increased significantly more than did the Ψ values in coding transcripts (*Fig. 4B*).

## DISCUSSION

**Fig. 5:**
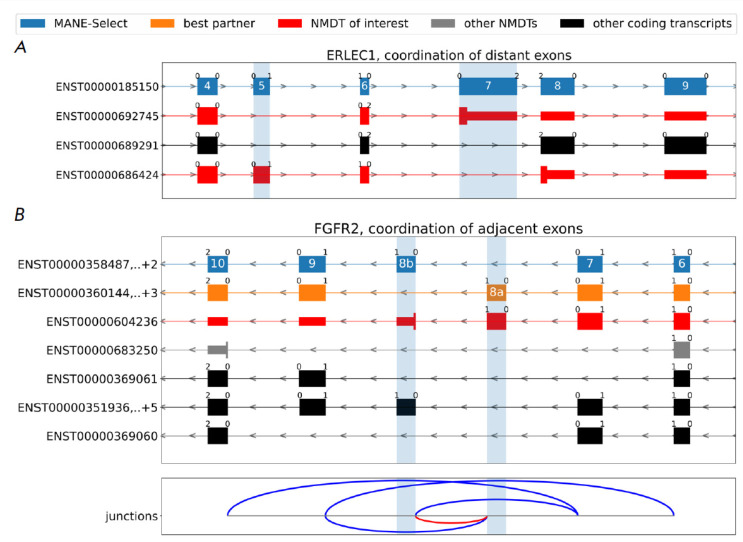
(A) Coordinated splicing of distant cassette exons in the ERLEC1 gene. (B) Coordinated splicing of adjacent exons in the FGFR2 gene. Besides mutually exclusive splicing of exons 8a and 8b, transcript isoforms with coordinated skipping of exons 7–9 (NMDT) and ones with coordinated skipping of exons 7–8a,b and 8a,b–9 (protein-coding) are annotated. Simultaneous inclusion of exons 8a and 8b generates NMDT. Legend colors are as in Fig. 2

The approach implemented in NMDj does not rely on a single best partner transcript, and that allows it to identify and properly describe many more NMDgenerating AS events as compared to NMD Classifier. However, NMDj was unable to locate characteristic introns for some NMDTs (1,139 transcripts), which in most cases was the result of coordination between distant AS events and the usage of alternative start and stop codons. For instance, in the *ERLEC1 *gene, simultaneous inclusion and exclusion of non-adjacent exons 5 and 7 preserves the reading frame, while inclusion of only one exon from the pair leads to NMDT (*Fig. 5A*). This example demonstrates that it is not always possible to establish a causal link between a particular local AS event and NMDT, because NMD sensitivity is a global property of a transcript which depends on coordination between distant AS events, while local AS events individually may not be capturing these global properties. Like other approaches that take into account only local AS events, NMDj is fundamentally incapable of correctly characterizing the cause of such NMDTs.

Local AS events are known to regulate gene expression by AS switching to NMDT production [5, 6]. Such switching is mediated by RNA-binding proteins that bind to the *cis*-elements in pre-mRNA and is typically regulated locally [37]. In contrast, little is known about the functional outcomes and exact regulatory mechanisms of coordination for AS events at large distances [38, 39, 40, 41]. While the coordination between distant AS events could be important for producing protein isoforms with distinct functions, in some cases cells could use it to generate NMDTs. An example of this is the coordinated, mutually exclusive splicing of exons 8a and 8b in the *FGFR2 *gene, which leads to functional protein products with different ligand specificities [41] (*Fig. 5B*). The inclusion of exon 8a is promoted by the epithelial-specific proteins ESRP1 and ESRP2, which bind to the same regulatory sequence inside the intron [42], but simultaneous inclusion of both exons generates NMDT. Thus, switching between FGFR2 isoforms is regulated on the level of local AS, while coordination of mutually exclusive exon choices is achieved by the elimination of an NMDT.

In sum, a simultaneous analysis of all splice isoforms, instead of single best-matching transcript partners, allows NMDj to identify NMD-generating AS events with higher accuracy. However, the technique shares a common limitation with other methods in classifying the coordinated action of distant AS events. Their analysis requires fundamentally different approaches. However, it seems more likely that NMD induces a non-random association of AS events than a regulated association of AS events induces NMD. Thus, the analysis of coordinated AS events falls outside the scope of this study for both technical and conceptual reasons.

The method developed in this paper can be used to study gene expression regulation via unproductive splicing [6]. In particular, it can be applied to problems such as searching for specifically expressed NMDTs and assessing the activity of the NMD system as a whole. Thus, NMDj closes the existing gap in the toolkit for studying the conjugation between AS and NMD.

## Abbreviations

NMD: Nonsense Mediated Decay
NMDT: NMD target transcript
PTC: premature termination codon
AS: alternative splicing
UTR: untranslated region
nt: nucleotide
